# Ultra-structural changes and expression of chondrogenic and hypertrophic genes during chondrogenic differentiation of mesenchymal stromal cells in alginate beads

**DOI:** 10.7717/peerj.1650

**Published:** 2016-03-01

**Authors:** Havva Dashtdar, Malliga Raman Murali, Lakshmi Selvaratnam, Hanumantharao Balaji Raghavendran, Abdulrazzaq Mahmod Suhaeb, Tunku Sara Ahmad, Tunku Kamarul

**Affiliations:** 1 Tissue Engineering Group, Department of Orthopaedic Surgery (NOCERAL), Faculty of Medicine, University of Malaya, Kuala Lumpur, Malaysia; 2 School of Medicine and Health Sciences, Monash University Malaysia, Sunway Campus Selangor, Malaysia; 3 Department of Orthopaedic Surgery, Faculty of Medicine, University of Malaya, Kuala Lumpur, Malaysia; 4 Clinical Investigative Centre (CIC), University Malaya Medical Centre, Kuala Lumpur, Malaysia

**Keywords:** Pellet culture, Alginate, Chondrogenesis, Mesenchymal stromal cells, Tissue engineering, Hypertrophic condensation

## Abstract

Chondrogenic differentiation of mesenchymal stromal cells (MSCs) in the form of pellet culture and encapsulation in alginate beads has been widely used as conventional model for *in vitro* chondrogenesis. However, comparative characterization between differentiation, hypertrophic markers, cell adhesion molecule and ultrastructural changes during alginate and pellet culture has not been described. Hence, the present study was conducted comparing MSCs cultured in pellet and alginate beads with monolayer culture. qPCR was performed to assess the expression of chondrogenic, hypertrophic, and cell adhesion molecule genes, whereas transmission electron microscopy (TEM) was used to assess the ultrastructural changes. In addition, immunocytochemistry for Collagen type II and aggrecan and glycosaminoglycan (GAG) analysis were performed. Our results indicate that pellet and alginate bead cultures were necessary for chondrogenic differentiation of MSC. It also indicates that cultures using alginate bead demonstrated significantly higher (p < 0.05) chondrogenic but lower hypertrophic (p < 0.05) gene expressions as compared with pellet cultures. N-cadherin and N-CAM1 expression were up-regulated in second and third weeks of culture and were comparable between the alginate bead and pellet culture groups, respectively. TEM images demonstrated ultrastructural changes resembling cell death in pellet cultures. Our results indicate that using alginate beads, MSCs express higher chondrogenic but lower hypertrophic gene expression. Enhanced production of extracellular matrix and cell adhesion molecules was also observed in this group. These findings suggest that alginate bead culture may serve as a superior chondrogenic model, whereas pellet culture is more appropriate as a hypertrophic model of chondrogenesis.

## Introduction

Chondrogenic differentiation is a unique process that starts with cell–cell interactions and is influenced by the presence of specific growth and differentiation factors. During embryonic limb formation, this process is described as condensation. Based on this phenomenon, different chondrogenic induction techniques have been developed to recapitulate the *in vivo* chondrogenesis with the hopes that tissue repair can be achieved in clinical applications. It has been shown that the use of monolayer cultures is inadequate to reproduce chondrogenesis and there is a need for three-dimensional (3D) culture systems in order for this to occur. As such, cultures using high density of cells in the form of pellet or aggregates or a combination of cells with biomaterials are used to provide a cell-embedded 3D structure. It is said that this produce highest cell–cell and cell–matrix interactions and responsible for cell differentiation process (Penick, Solchaga & Welter, 2005). However, in addition to inducing chondrogenesis, most of these culture conditions also lead towards a hypertrophic differentiation after the initial chondrogenic induction, similar to that observed in the terminal differentiation of hypertrophic chondrocytes during endochondral ossification (Ma et al., 2003; Steinert et al., 2003; Ichinose et al., 2005; Xu et al., 2008; Bian et al., 2011). This unstable chondrogenic phenotypic expression after the initial induction was considered to be the major hurdle to *in vitro* chondrogenesis for application in cartilage tissue engineering.

During the initial stages of chondrogenesis and condensation of mesenchymal stromal cells (MSCs) in limb bud formation, higher expressions of two major cell adhesion molecules N-CAM and N-cadherin have been reported (Monroy & De Leon, 1999; Woodward & Tuan, 1999; Hall & Miyake, 2000). These molecules, however, appear to be downregulated after chondrogenic differentiation and only expressed in the periphery of the limb anlagen *in vivo* or chondrogenic aggregate *in vitro* (Widelitz et al., 1993; Tavella et al., 1994). In articular cartilage, each chondrocyte has been shown to be responsible for the production of extracellular matrix (ECM), which, when fully formed, creates a functional unit of cartilage or chondron that ultimately leads to the formation of cartilage matrix (Poole, 1997) and there appears no direct cell–cell contact. Chondrocyte serves as the only responsible cell type for tissue homeostasis or synthesis and degradation of ECM (Pearle, Warren & Rodeo, 2005); therefore, a profound understanding of the morphology and physiology of the engineered chondrocyte-like cell is required to determine the likelihood of cartilage regeneration outcomes.

Although alginate bead culture system has been shown to provide beneficial microenvironment for evaluating chondrogenesis of MSCs *in vitro* (Yang et al., 2004; Ichinose et al., 2005; Xu et al., 2008; Duggal et al., 2009; Diekman et al., 2010), it has not been studied in greater detail. This is especially true when determining the cell adhesion molecules, hypertrophic genes, and detailed ultrastructural studies relating to cell–matrix interactions during the chondrogenic differentiation of MSCs in alginate beads. In addition, the advantage of one group over (pellet culture vs alginate culture) the other has not been previously demonstrated. Therefore, the present study was conducted to examine chondrogenic, hypertrophic, and cell adhesion molecule gene expressions and ultrastructural changes of chondrogenic differentiated MSCs in alginate beads and compare them with pellet and monolayer cultures.

## Materials and Methods

### Isolation and characterization of human bone marrow stromal cells

Human bone marrow samples were obtained from healthy adults (male, age =21 + 2.6 years) who were undergoing fracture fixation involving the long bones. After providing the patient information sheet and explaining the patients on bone marrow collection, written informed consent was obtained and bone marrow was collected. This study was approved by the University of Malaya Medical Center Ethics Committee (reference no. 602.22). Human bone marrow was collected (*N* = 6) in sterile 3-ml BD Vacutainer blood tubes (K2 EDTA, BD, Franklin Lakes, NJ, USA) by orthopedic surgeons and was kept at 4 °C until isolation. The mononuclear cells were isolated using Ficoll density gradient method as described earlier (Krishnamurithy et al., 2014). Isolated cells were cultured and expanded until P3 in T-75 flasks. The isolated cells were characterized as MSCs through flow cytometry using cell surface markers (positive and negative markers) and its ability to undergo trilineage differentiation (adipogenic, chondrogenic, and osteogenic differentiation) was described earlier (Tan et al., 2013). MSCs isolated form three biological samples were used for gene expression analysis and three biological samples were used for imaging analysis.

### Chondrogenic differentiation and experimental groups

Pellet culture: MSCs were harvested at P3, and 2.5 × 10^5^ cells were pelleted in a 15-ml propylene centrifuge tube at relative centrifugal force (RCF) of 260 × g 5 min. After removal of the supernatant, the pellet cultured with 2 ml of chondrogenic medium contains the following: DMEM high glucose (4.5 mg/ml d-glucose) with sodium pyruvate (110 µg/ml) (Invitrogen, Carlsbad, CA, USA), 50 mg/ml ITS (Sigma) (1×) (Invitrogen), l-ascorbate 2 phosphate (50 µg/ml) (Sigma, St. Louis, MO, USA), 10 ng/ml TGF*β*3 (Invitrogen), 100 nM dexamethasone (1 × 10^−7^ M) (Sigma), 100 µg/ml penicillin/streptomycin (Invitrogen), and 40 µg/ml l-proline (Sigma). The media were changed every 3 days.

Alginate cell constructs: 1.2% alginate prepared from alginic acid powder, low viscosity (Sigma-Aldrich) in 0.9% sodium chloride (NaCl) and filtered sterile by a 0.2-µm filter. MSCs at P3 were harvested, and a concentration of 4 × 10^6^ cells per milliliter of alginate was obtained before dropping into sterile calcium chloride solution (CaCl_2_) using a pipette. Alginate bead constructs were cross-linked in this solution for 10 min in an incubator at 37 °C, were rinsed in 0.9% normal saline 2–3 times, and then transferred to the culture dishes (ultra-low attachment 6-well plates; Corning, New York, NY, USA). Three beads were placed per well (about 80,000 cells per bead) and supplemented with 2 ml of chondrogenic medium. The medium was changed every 3 days. Alginate cell constructs were dissociated in a buffer solution containing 0.015 M sodium citrate and 0.15 M sodium chloride (Na_3_C_6_H_5_O_7_ dehydrate 2H_2_O, MW = 294.10), pH 7.2, and centrifuged at 1,100 rpm for 5 min before gene expression studies were conducted.

Monolayer culture: MSCs at P3 were cultured in chondrogenic medium at a density of 4,000 cells per centimeter in 6-well plates.

### Glycosaminoglycan (GAG) analysis and DNA quantification

Alginate beads and pellets from days 3, 12, and 21 were washed with phosphate-buffered saline. Alginate bead was dissolved in sodium citrate and the pellets were crushed and the samples were digested in papain buffer overnight at 60 °C. The digested samples were subjected to biochemical analyses to determine the glycosaminoglycan and DNA content. GAG production was determined by using Blyscan assay kit (Biocolor, arrickfergus, Northern Ireland), according the manufacturer’s protocol. GAG content was determined using a standard curve drawn using standard solutions containing chondroitin 4-sulfate. Alginate beads without cells at the respective time point was analyzed in the same manner and the values are used as blank. DNA content was determined by using the fluorescent picoGreen dsDNA quantification assay (Invitrogen). GAG in alginate bead and pellet culture system was expressed as µg GAG per µg of DNA.

### Morphological studies

On day 21, samples were fixed in 10% formalin and processed for histological studies stained with Safranin O Fast green or immunohistochemistry for Collagen type II (Mouse monoclonal anti-Human; Merk, Darmstad, Germany) in 1/100 dilution and aggrecan (Mouse monoclonal anti-Human; Abcam, Cambridge, UK) in 1/50 dilution using EnVision+ System-HRP (DAB) Dako kit (Dakocytomation, Glostrup, Denmark) according to the manufacturer’s protocol.

### RNA isolation and cDNA synthesis

Total RNA was isolated from cultures of pellet, alginate, and monolayer on days 3, 12, and 21. RNA was isolated using SV total RNA isolation system (Promega, Madison, WI, USA) according to the manufacturer’s protocol. One hundred nanograms of RNA was used to generate cDNA with iScriptTM Reversed Transcription Supermix for RT-qPCR (BioRad) according to the manufacturer’s protocol.

### Quantitative real-time polymerase chain reaction

PCRs were carried out in duplicate for each three biological sample in a final volume of 20 µl containing SYBR Green mastermix (BioRad, Hercules, CA, USA) with 160 nM concentration of each primer and 100 ng cDNA in 0.2-ml PCR tubes using CSFX96TM Real Time System, Bio-Rad, under the following conditions: 3 min at 95 °C followed by 40 cycles at 58.6 °C for 0.20 s as annealing temperature and 72 °C for 0.30 s as extension. The reactions were ended by 0.1 min at 95 °C and a melt curve by increasing temperature from 65 °C, 0.05 min, to 95 °C, 0.5 min, stepwise. No template controls were used in each reaction as negative control. The data were presented as a time fold change relative to the internal control gene expression. The data were then normalized to transcription levels of day 0 culture using ΔCT and ΔΔCT methods. Values below 1 were considered downregulated. The following primer sets were applied in this experiment (Table 1).

**Table 1:** Primer sequence.

Gene	Access no.		Primer pairs 5′-3′	Amplicon size/reference
Aggrecan	NM_001135.3	F	CTACGACGCCATCTGCTACA	141
		R	TCAGTGATGTTTCGAGGCAG	
Beta-actin	NM_001101.3	F	CTCTTCCAGCCTTCCTTCCT	116
		R	AGCACTGTGTTGGCGTACAG	
Collagen II	NM_033150.2	F	GAAAGCCTGGTGATGATGGT	138
		R	GGCCTGGATAACCTCTGTGA	
Collagen X	NG_008032.1	F	CACCTGTGGTCCTGAATGTG	163
		R	TCTGAGTGCCTGGATGTCTG	
N-Cadherin	NM_001792.3	F	GGAAAAGTGGCAAGTGGCAG	159
		R	GGAGGGATGACCCAGTCTCT	
NCAM1	NM_000615.6	F	AGGAGACAGAAACGAAGCCA	161
		R	GGTGTTGGAAATGCTCTGGT	
Run X2	NM_001015051.3	F	TTACTTACACCCCGCCAGTC	139
		R	CACTCTGGCTTTGGGAAGAG	
SOX9	NM_000346.3	F	AGACAGCCCCCTATCGACTT	108
		R	CGGCAGGTACTGGTCAAACT	
GAPDH	Bian et al. (2011)	F	AGGGCTGCTTTTAACTCTGGTAAA	111
		R	GAATTTGCCATGGGTGGAAT	

### Transmission electron microscopy (TEM)

Samples for TEM study were fixed with 4% glutaraldehyde in cacodylate buffer for 24 h at 4 °C and postfixed in buffered 1% osmium tetra oxide for 2 h at 4 °C, washed with cacodylate buffer 2–3 times, and kept overnight at 4 °C in cacodylate buffer before en bloc staining with 4% uranyl acetate (Agar Scientific Ltd R1043, Essex, England) in double distilled water for 10 min. Samples were then dehydrated in ascending series of ethanol and embedded in Epon (Agar 100 resin kit; Agar Scientific Ltd R1043).

Semi-thin 1 µm sections were obtained using a Leica ultramicrotome (Reichert Ultracuts; Leica Microscystem, Vienna, Austria) and stained with toluidine blue for 1–2 min before viewing with light microscopy. Ultrathin sections were cut at 70–80 nm, collected on copper grids (300 meshes), and stained with uranyl acetate and lead citrate (Agar Scientific). Images were viewed using TEM (Leo Libra 120; Carl Zeiss SMT AG, Oberkochen, Germany).

### Statistical analysis

The difference between experimental groups was calculated using nonparametric Kruskal-Wallis *H* test and the difference between two independent experimental groups using Mann–Whitney *U* test, available on the statistical software package SPSS (version 18.0), with *p* ≤ 0.05 being considered significant.

## Results

### Chondrogenic differentiation

**Figure 1:**
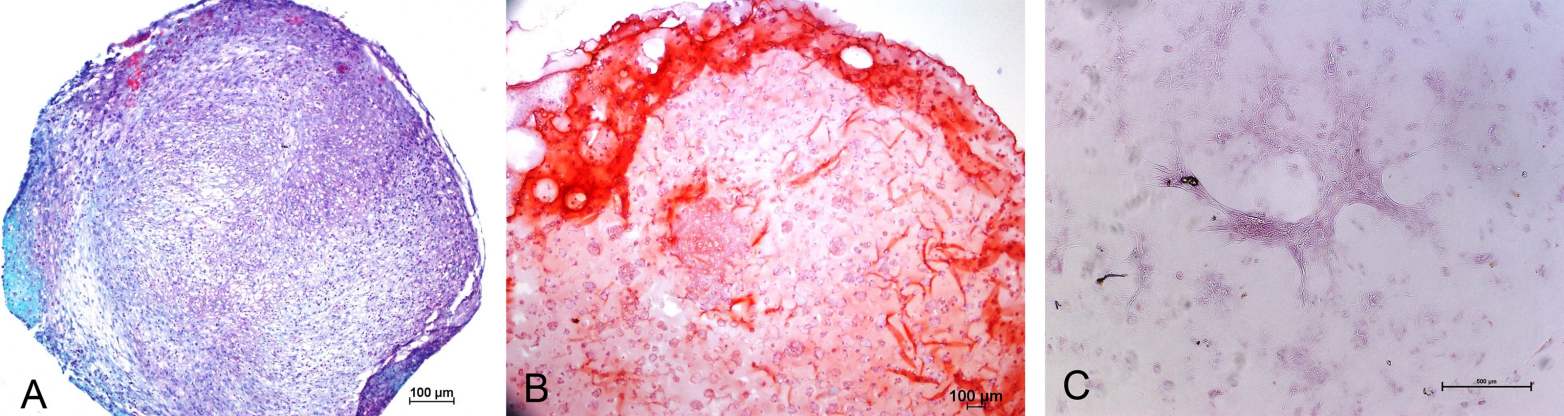
Safranin O Fast green staining of MSCs. Images showing GAG production in Pellet culture (A), Alginate beads (B) and Monolayer (C) for 21 days (10 X).

Chondrogenic differentiation of MSCs in different experimental groups was shown in Fig. 1. Safranin O Fast green staining of pellet culture (Fig. 1A) on day 21 showed red staining positive for GAG and green area indicating non-GAG depositions. In alginate culture on day 21, Safranin O Fast green showed intense staining of red color (Fig. 1B), indicating high GAG production in this group compared with control group (Fig. 1C).

### Immunohistochemistry for Collagen type II and aggrecan

**Figure 2:**
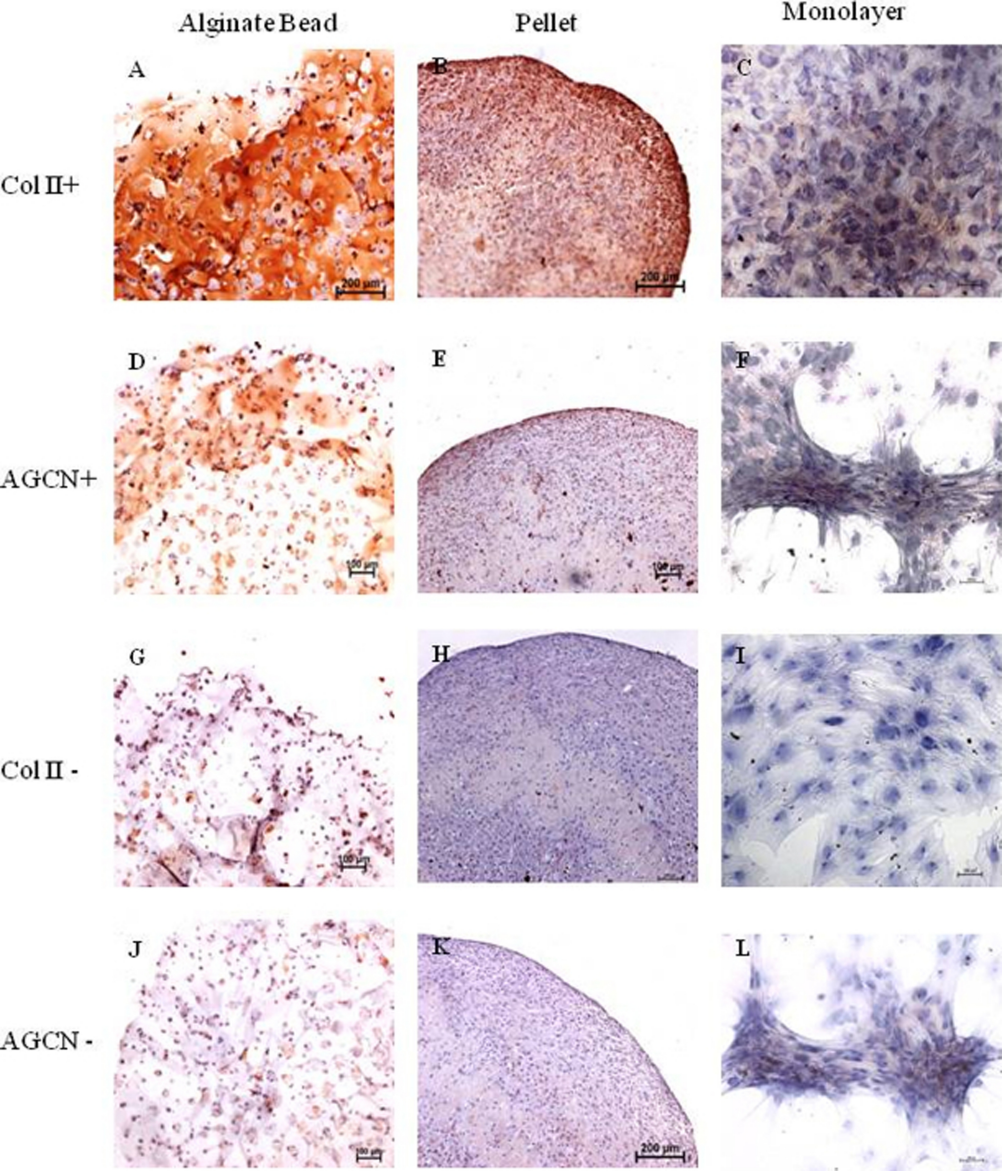
Immunohistochemistry of Collagen II and Aggrecan. Immunohistochemistry of Collagen II (A)–(C) and Aggrecan (D)–(F) in Alginate bead, pellet culture and monolayer after 21 days of culture in chondrogenic medium. (G)–(I) and (J)–(L) represents negative control for Collagen II and Aggrecan.

Monolayer, pellet, and alginate bead cultures showed positive for Collagen type II staining on 21 days of culture (Figs. 2A–2C), compared with the negative controls (Figs. 2G–2I). Immunochemistry of aggrecan showed positive for pellet and alginate bead cultures (Figs. 2D–2E); however, no positive staining was observed in monolayer (Fig. 2F) compared with the negative control (Figs. 2J–2L).

### Glycosaminoglycan/DNA quantification

**Figure 3:**
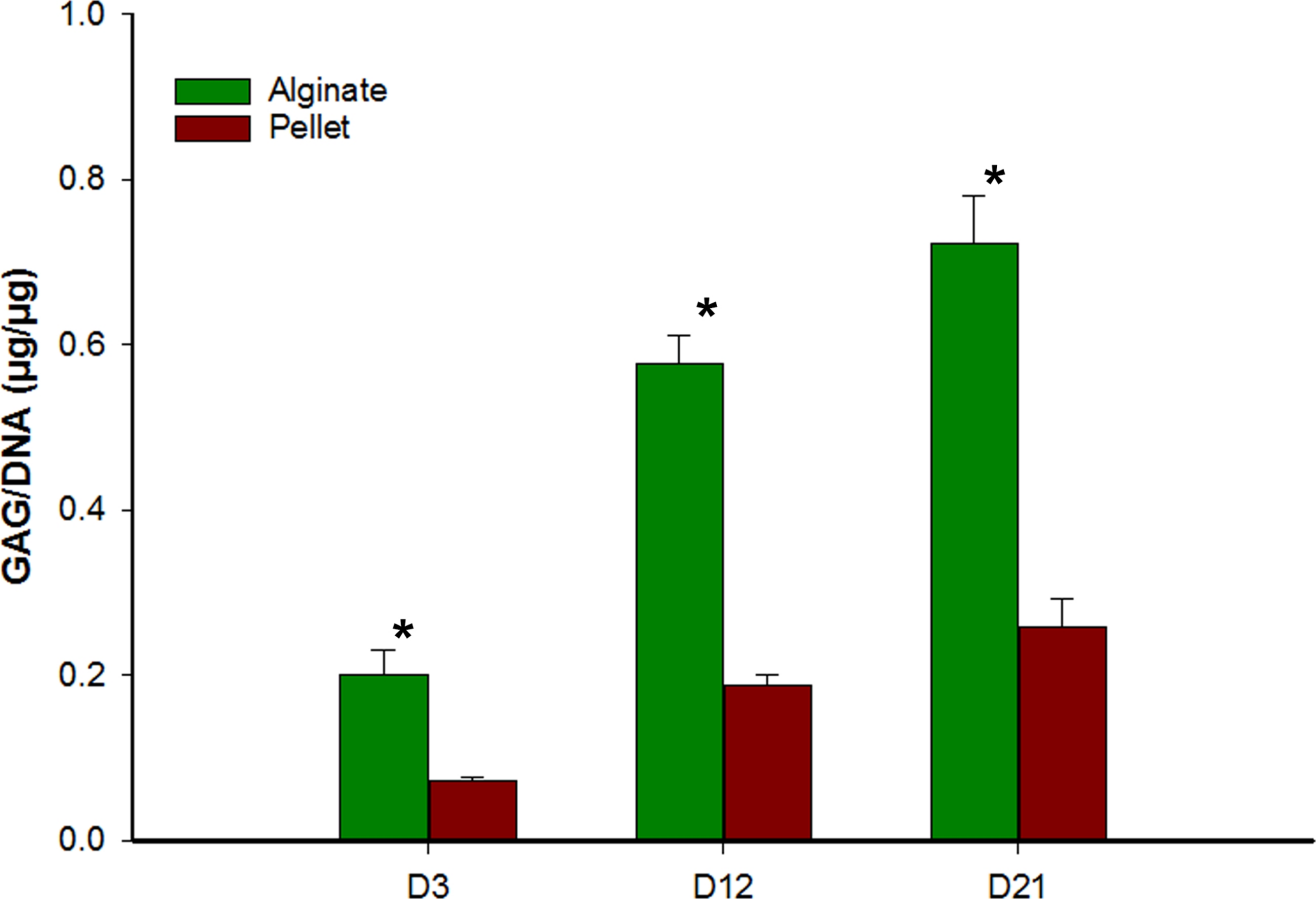
Comparison of glycosaminoglycan content from alginate bead and pellet culture system. GAG content in the alginate bead and pellet culture system was normalized to respective DNA content. Data shown are mean ± SD. ^∗^ − *p* < 0.05.

Figure 3 represents the GAG/DNA content of alginate bead and pellet cultures. The results indicate that hMSCs differentiated in alginate beads has produced significantly higher (*p* < 0.05) glycosaminoglycan than cells in the pellet culture system. The increase in GAG content was also significant over time, day 3 to day 12 and day 12 to day 21, in both alginate bead and pellet culture.

### Chondrogenic gene expression

**Figure 4:**
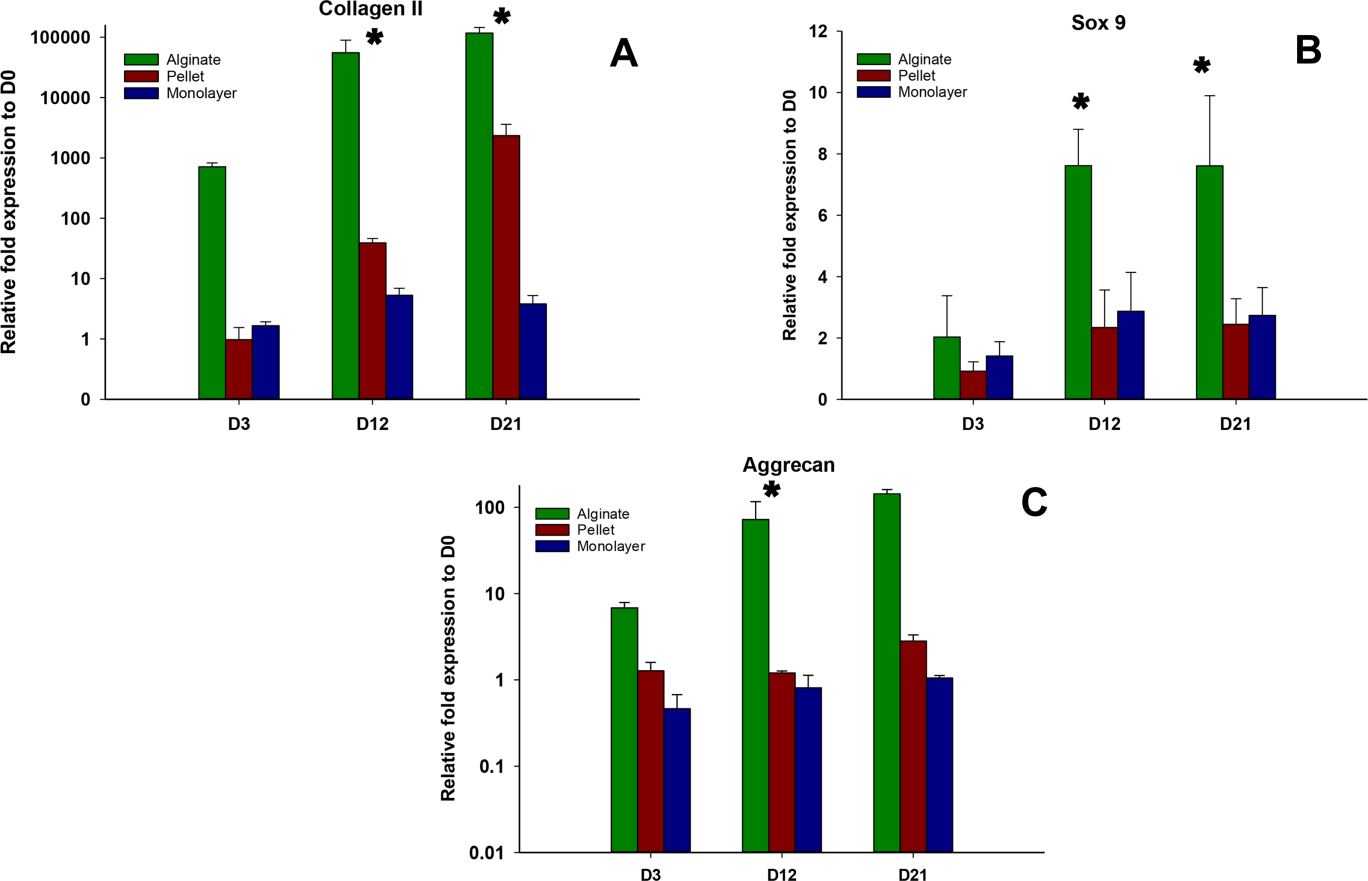
Expression of Chondrogenic genes. Gene expression of Collagen II (A), Sox 9 (B) and Aggrecan (C) in alginate, pellet and monolayer cultures at day 3, day 12 and day 21.

Expressions of chondrogenic genes, Collagen II, Sox 9, and aggrecan, were shown in Figs. 4A–4C, respectively. In all time points (days 3, 12, and 21), alginate culture represents significantly higher (*p* < 0.05) chondrogenic gene expression compared with monolayer and pellet cultures. In alginate group, there was a significant increase between days 3 and 12 and no significant increase was observed between day 12 and 21 for Collagen II and Sox 9, whereas no significant increase was observed between days 12 and 21 for aggrecan. For pellet culture, there was a significant increase from days 3 to 21 for Collagen II and Sox 9 and expression of Sox 9 and aggrecan remains non-significant from day 12 to 21.

### Hypertrophic gene expression

**Figure 5:**
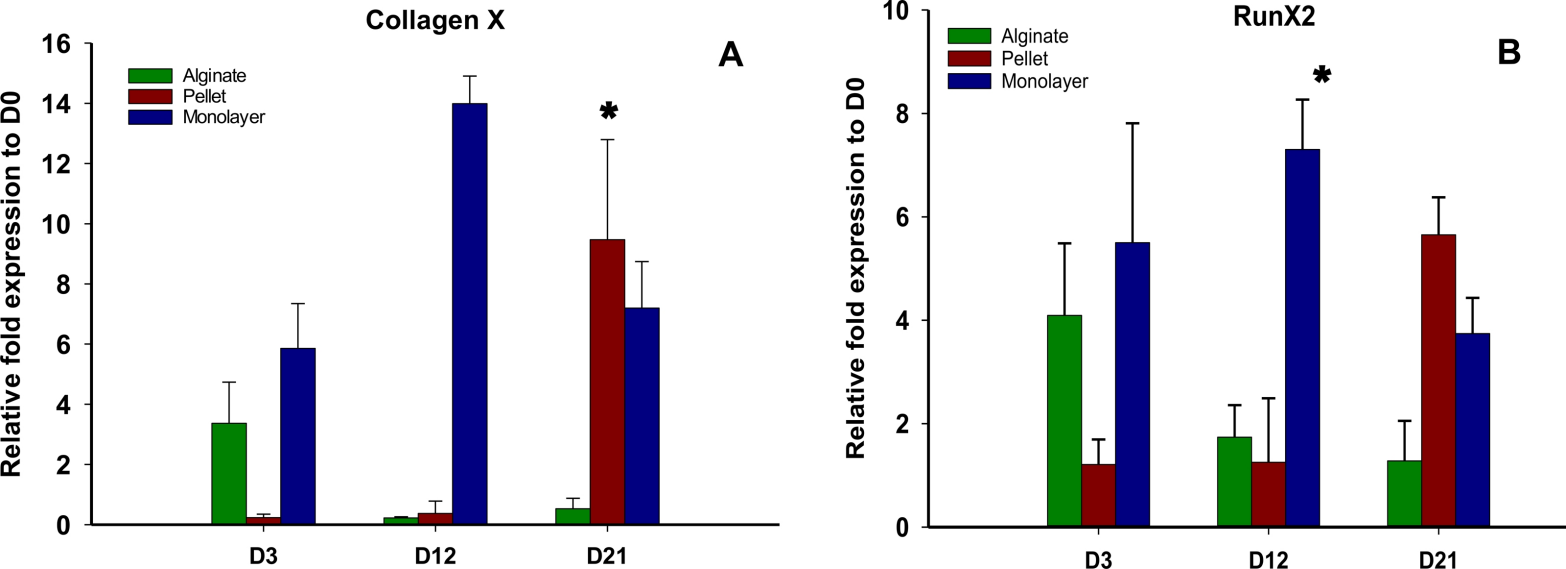
Expression of Hypertrophic genes. Gene expression of Collagen X (A) and Run X2 (B) in alginate, pellet and monolayer cultures at day 3, day 12 and day 21.

The expressions of hypertrophic genes Collagen X and Runx2 are shown in Figs. 5A and 5B, respectively. The expressions of these genes were significantly higher (*p* < 0.05) in alginate beads at the earlier time point (day 3) compared with pellet culture; on the other hand, these genes were found to be significantly down regulated in the later time points of days 12 and 21. At day 3 no significant difference was observed between the expression of these genes between monolayer and alginate bead culture. In pellet culture, although the expressions of hypertrophic genes were significantly lower on day 3, the expression level was significantly increased over time.

### Cell adhesion molecule expression

**Figure 6:**
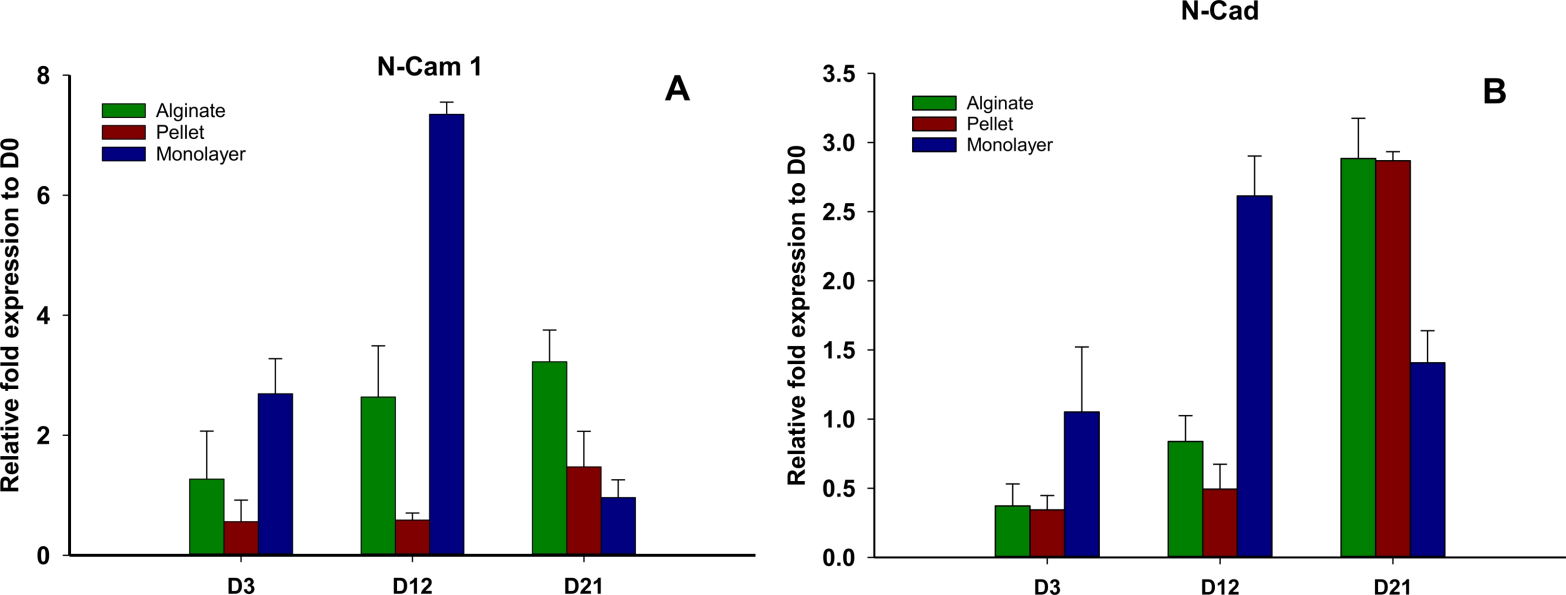
Expression of cell adhesion molecules. Gene expression of N-CAM1 (A) and N-Cadherin (B) in alginate, pellet and monolayer cultures at day 3, day 12 and day 21.

N-CAM1 and N-cadherin (Figs. 6A and 6B) remained downregulated on days 3 and 12 in pellet culture and upregulated on day 21, but in alginate group, N-CAM1 was upregulated over time from days 3 to 21, and for alginate and pellet cultures, N-cadherin remained downregulated on days 3 and 12 but is upregulated on day 21. In monolayer, the expression of N-CAM1 was not significant on days 3 and 12, whereas it was seen upregulated on day 21 and N-cadherin was increased significantly over time.

### TEM of MSCs in pellet and alginate cultures

**Figure 7:**
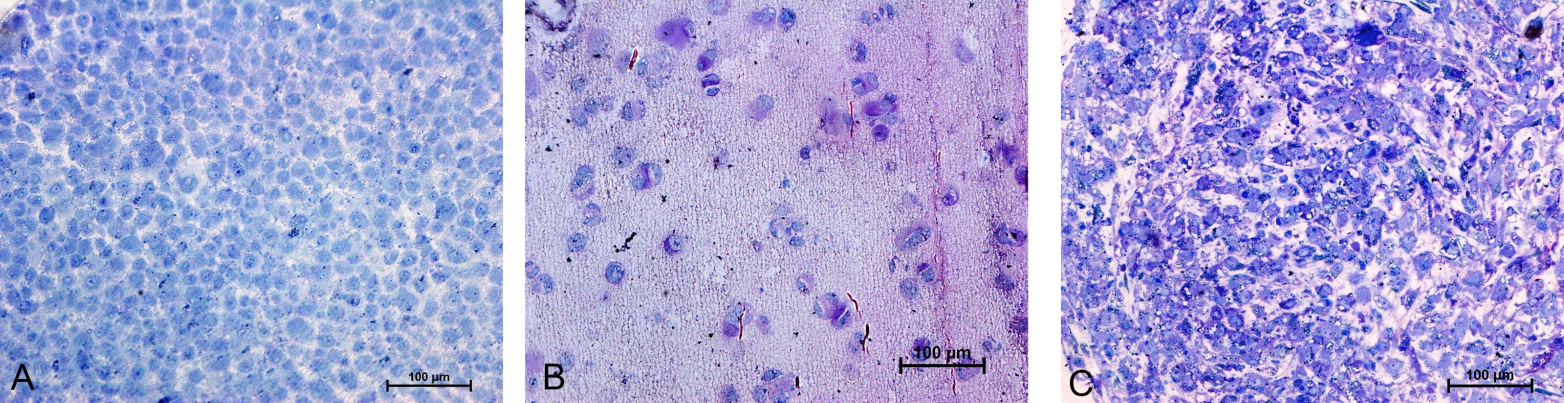
Toluidine blue staining of semi-thin sections (1 µm) of Alginate bead (B) and pellet culture (C) after 21 days of culture in chondrogenic medium and MSCs in day 0 (A). 20X.

**Figure 8:**
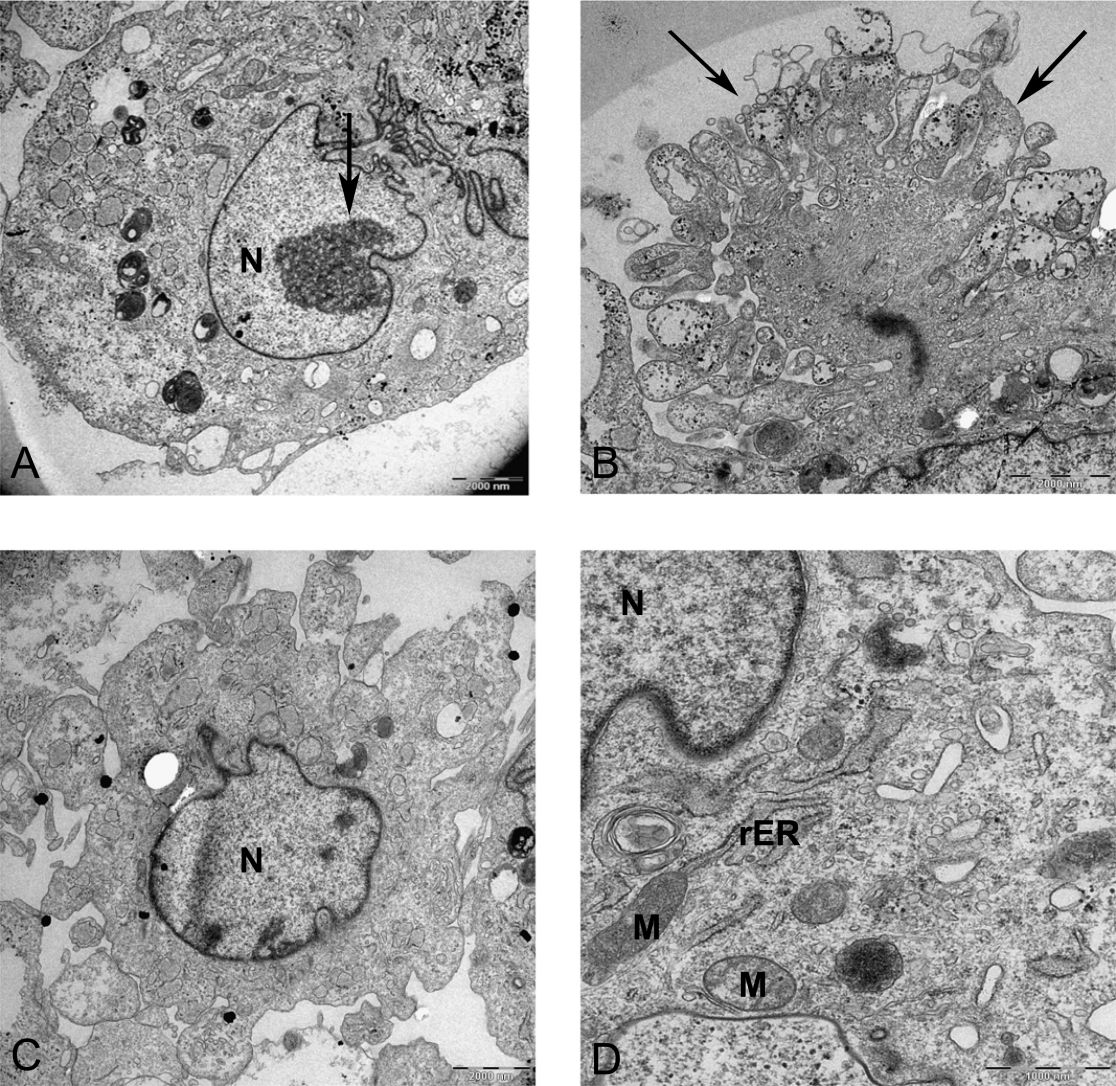
Transmission electron microscope images of mesenchymal stromal cells at day 0. TEM images of MSCs day 0. (A) MSC with a convoluted nucleus and prominent nucleoli (arrow) 1151X. (B) Blebs in the cell surface (arrow) 1600X N, Nucleus. (C) MSC with a round nucleus and uneven cell surface 1233X. (D) Higher magnification of MSCs shows mitochondria (M) and rough endoplasmic reticulum (rER) 4000X.

Resin-embedded samples of pellet and alginate cultures on day 21 and MSCs on day 0 were cut into semithin 1-µm sections, stained with toluidine blue, and studied under light microscopy (Fig. 7). Ultrathin sections of 70 nm thickness on copper grids were cut, stained with uranyl acetate and lead citrate, and studied with electron microscopy (Figs. 8 and 15).

The prominent features of semithin sections of alginate beads compared with pellet culture were lower cellularity and higher intercellular spaces (Fig. 7B). In alginate culture, cells were arranged in small groups of two or three cells per group and the matrix between them was stained purple using toluidine blue (Fig. 7B), whereas in pellet culture, cells were in closer contact with each other and less purple color indicated lower extracellular deposition (Fig. 7C).

The presence of abundance of polyanions in the ECM of cartilage gives a purple color to the metachromatic dye such as toluidine blue. Samples of MSCs on day 0 as a control group stained blue, whereas samples of chondrogenic differnetiated mesenchymal stromal cells (CMSCs) in alginate and pellet cultures showed positive metachromatic areas (purple color). However, no difference was observed between alginate and pellet culture and this might be due to the limitation of ultra-thin sections used.

### Ultrathin sections of pellet and alginate beads containing chondrogenic MSCs

In monolayer, MSC surface formed filopodia or blebs associated with dense bodies (Figs. 8B and 8C) and cytoplasm occupied with rER, free ribosomes, mitochondria (M), and vacuoles (Fig. 8). Nuclei contains euchromatin, usually with a distinct nucleolus, demonstrating an active protein-synthesizing cell; in some cells, the nucleus was convoluted (Fricker et al., 1997) (Fig. 8A).

**Figure 9:**
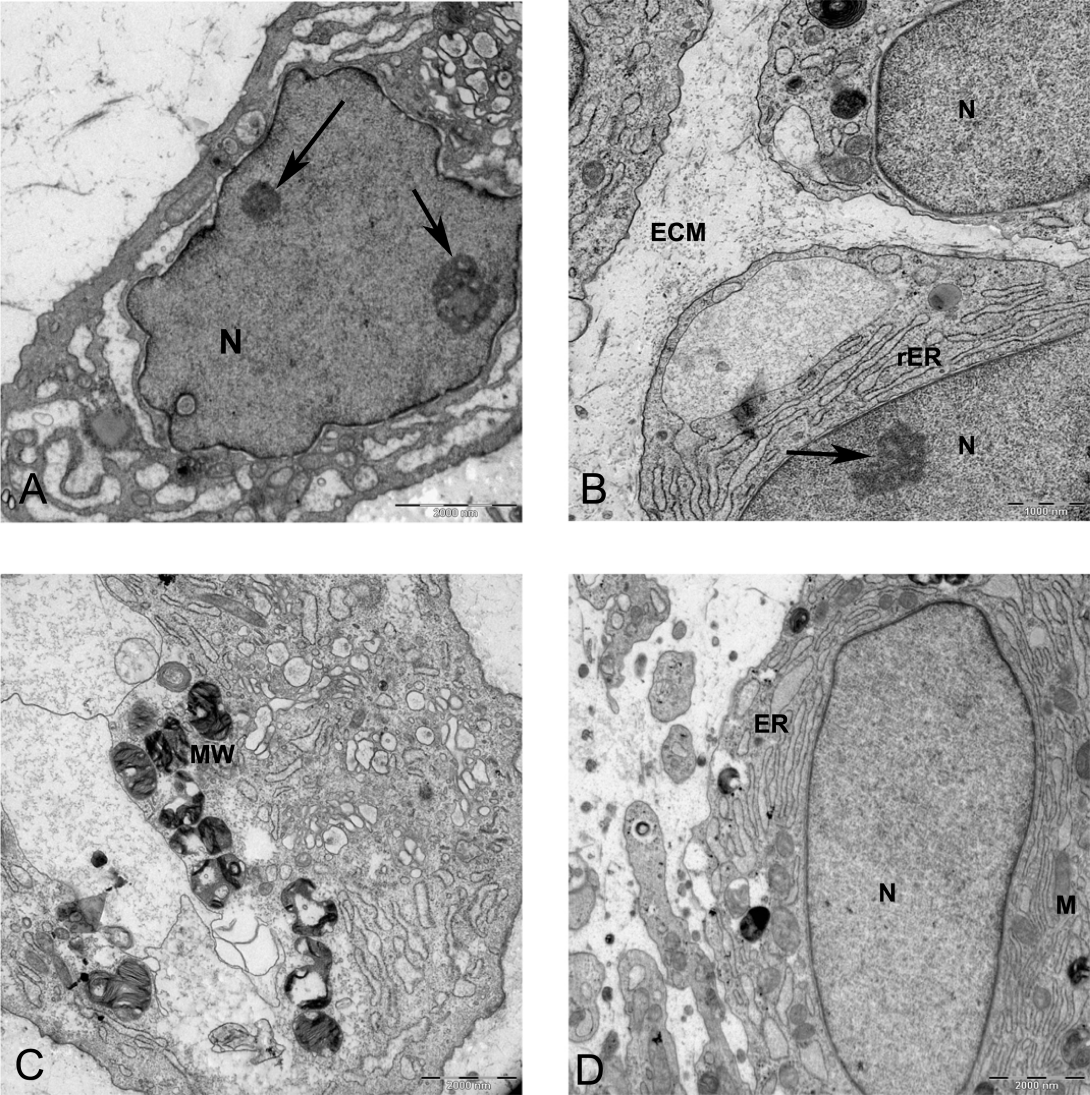
TEM images of MSCs differentiated in alginate bead after 21 days in chondrogenic medium. (A) The cytoplasm is filled with distended RER, Golgi apparatus in the supra-nuclear region (G) 2000X. (B) Three cells similar to isogenic groups in cartilage deposit their products in the 2520X. (C) A cell undergoing cell death with no clear nucleus and abundant cytoplasmic vesicles (V) and cytoplasmic inclusions or multilayer whorled membrane (MW) 1575X. (D) A cell with a euchormatic nucleus, rER and mitochondria (M) 1575X. N, Nucleus; RER, Rough endoplasmic reticulum; ECM, Extracellular matrix.

**Figure 10:**
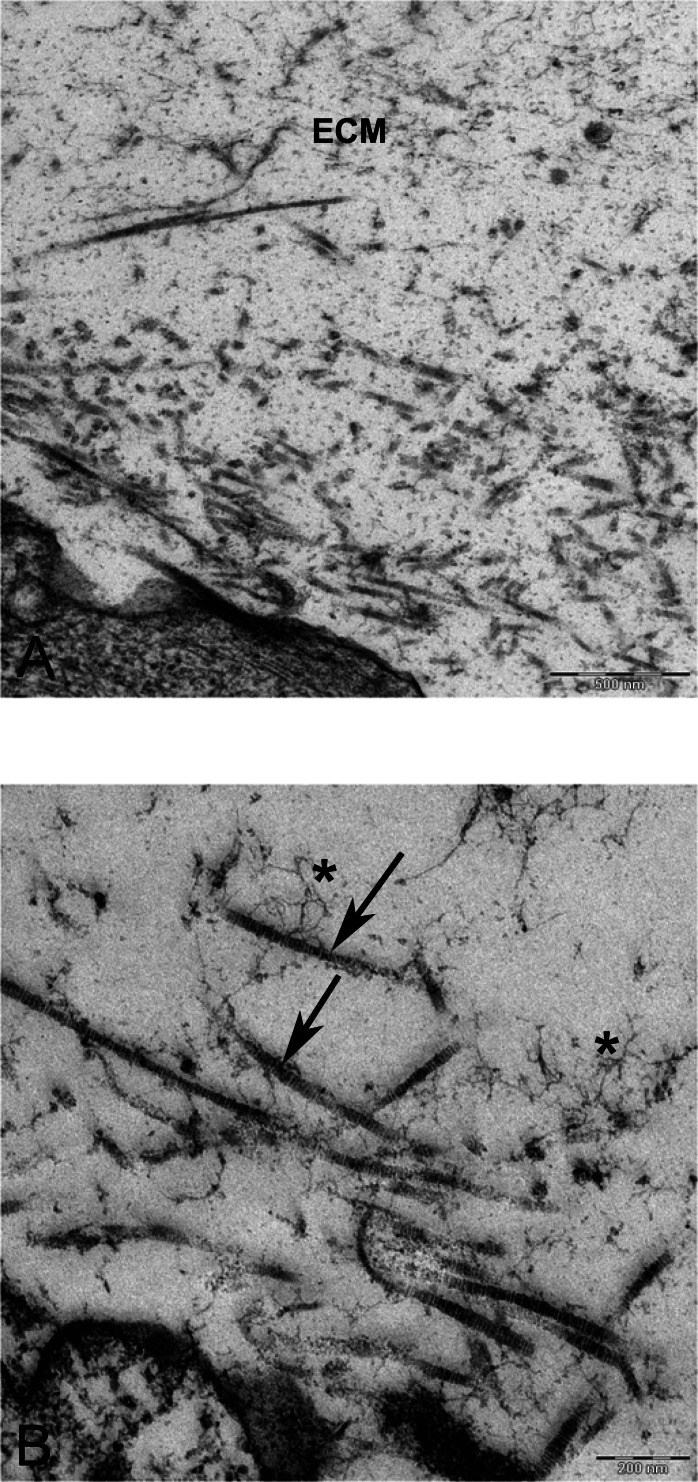
TEM images of extra cellular matrix of MSCs differentiated in alginate bead after 21 days in chondrogenic medium. (A) Extracellular matrix in CMSC. ECM, Extracellular matrix; C, Cell 6300X. (B) Higher magnification shows striation of collagen fibres (arrow) and branched molecules probably Aggrecan (asterisk) 10000X.

**Figure 11:**
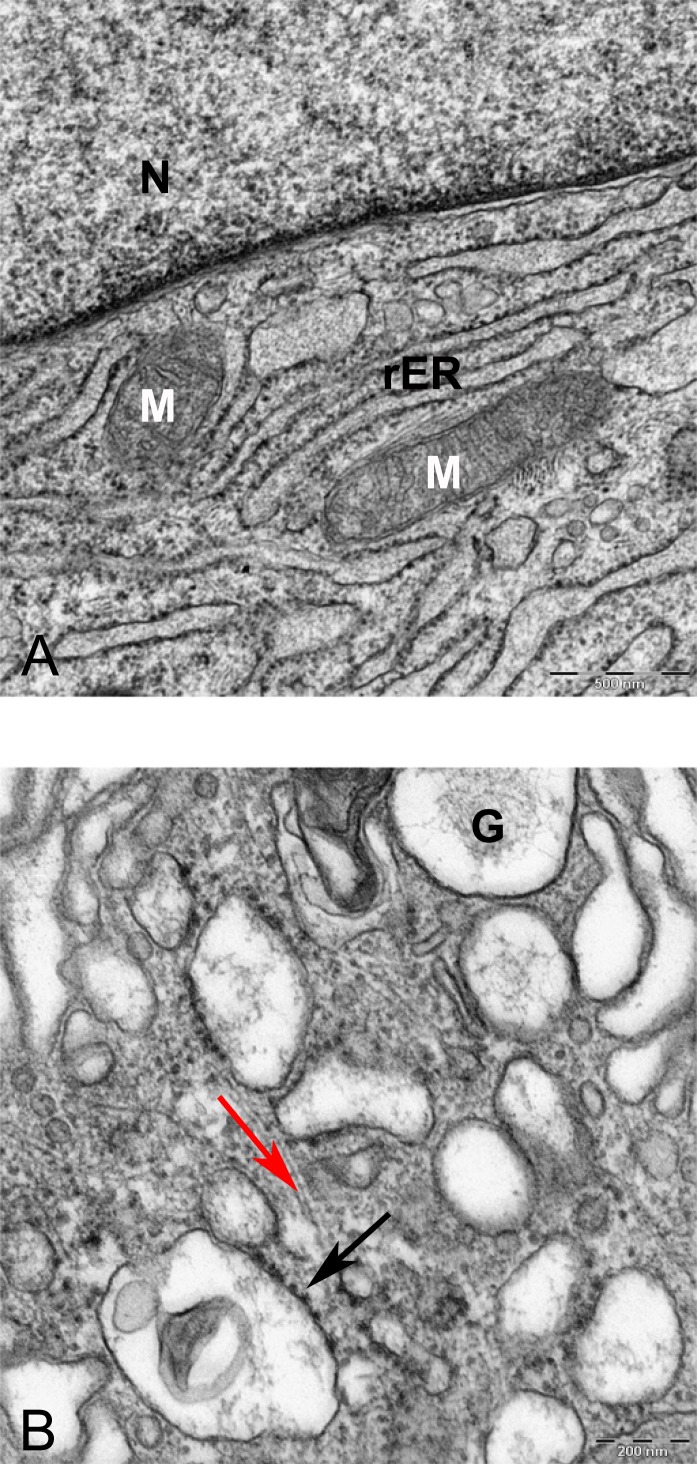
TEM image of perinuclear cytosol of MSCs differentiated in alginate bead after 21 days in chondrogenic medium. (A) A part of nucleus (N) and cytoplasm containing rough endoplasmic reticulum (rER) and Mitochondria (M) 6300X. (B) High magnification of cytoplasm. G, Golgi, black arrow indicates ribosomes, Red arrow shows microtubules, rER, Rough endoplasmic cytoplasm 10000X.

**Figure 12:**
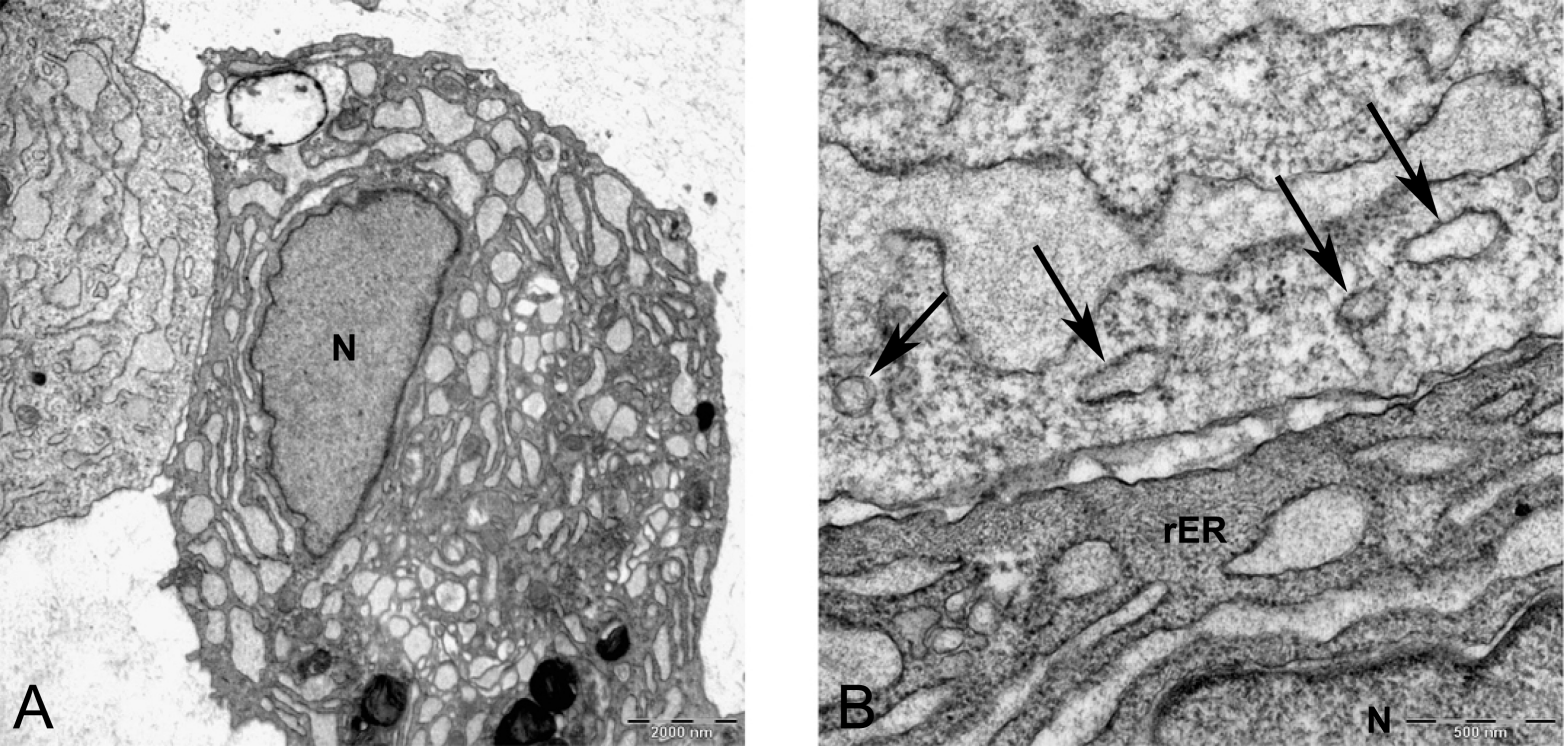
TEM image of divided cells in alginate bead after 21 days in chondrogenic medium. (A) Two daughter cells resulted of cell division 1260X. (B) Higher magnification of the inset shows ECM (asterisk) in the inter-cellular space. Note small vesicles near cytoplasmic membrane (arrows), rER, rough endoplasmic reticulum 6300X.

In alginate bead culture, ultrastructure of nucleus in CMSC showed euchromatin with a variety of phenotypes, round, oval, or slightly indented (Figs. 9 and 12). Chondrogenic alginate shows active protein synthesis, with euchromatic nuclei, prominent nucleoli, and abundant rER filled with electron lucent materials producing abundant ECM, containing collagen fibers (Figs. 9, 10 and 12) and molecules with side branches similar to proteoglycan aggregates among fibers (Fig. 10B). Chondrogenic differentiated cells are arranged in small groups as they were seen in lower magnification in semithin (Fig. 7B) and ultrathin sections (Figs. 9B and 12A). Higher magnification of adjacent cells did not show any junctional complexes between the cells. Smaller vesicles contained electron lucent material near cell membrane (Fig. 12A) that might originate from rER releasing their content to ECM, causing the cells grow apart from each other, similar to that seen during the interstitial growth in cartilage. The ECM was rich in collagen fibers with banding patterns (Fig. 10). In active protein synthesis, chondrogenic cells in alginate were difficult to distinguish between distended rER and Golgi apparatus (Fig. 9A); however, in higher magnification, the rER can be easily recognized with studded ribosomes (Fig. 11B, black arrow).

**Figure 13:**
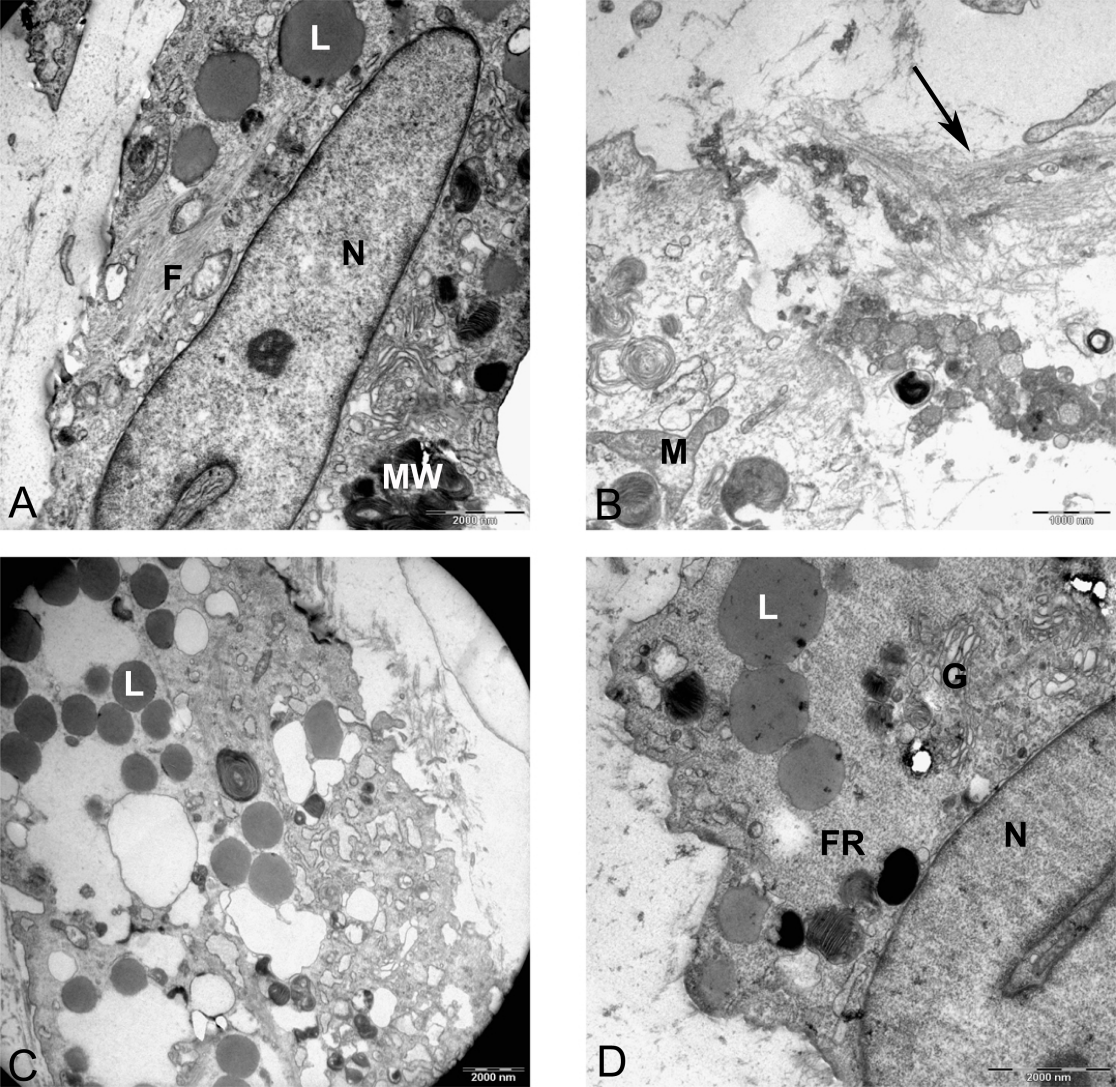
TEM images representing cell death in pellet culture after 21 days in chondrogenic medium. (A) Increase of lipid droplet, N, nucleus; MW, Multivesicular membrane; L, lipid droplet; M, mitochondria; F, cytoplasmic fibrils, 1600X. (B) Fused mitochondria (M), expelled cell organelles including mitochondria (arrow) 2520X. (C) No clear nucleus, 1000X. (D) Cell with U-shape nucleus, free ribosomes (FR) in cytoplasm, Golgi apparatus (G), and lipid droplets (L) 1984X.

**Figure 14:**
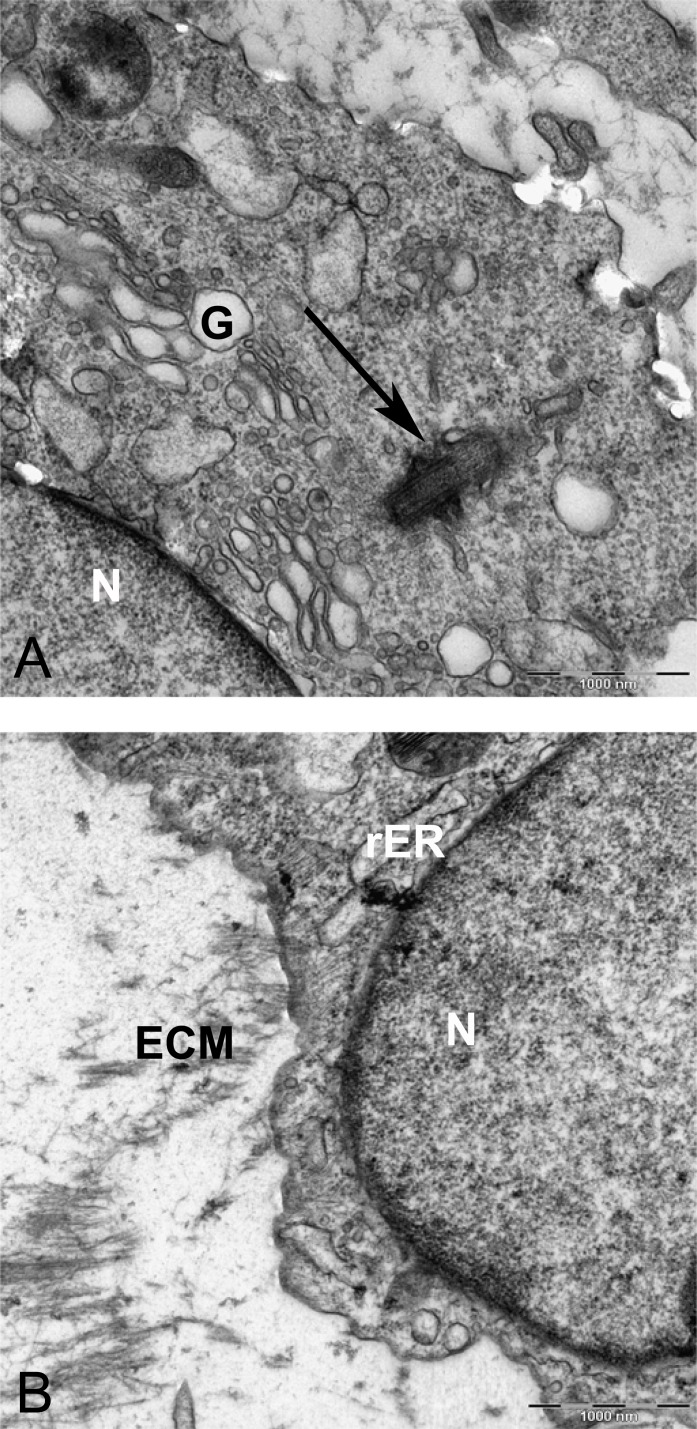
TEM image of MSCs in pellet culture after 21 days in chondrogenic medium. (A) Well-defined Golgi apparatus and Centriole (arrow) in the perinuclear cytosol 4000X. (B) Collagen fibers (F) secreted to the ECM directly from the cytoplasm (white arrow) 3969X.

**Figure 15:**
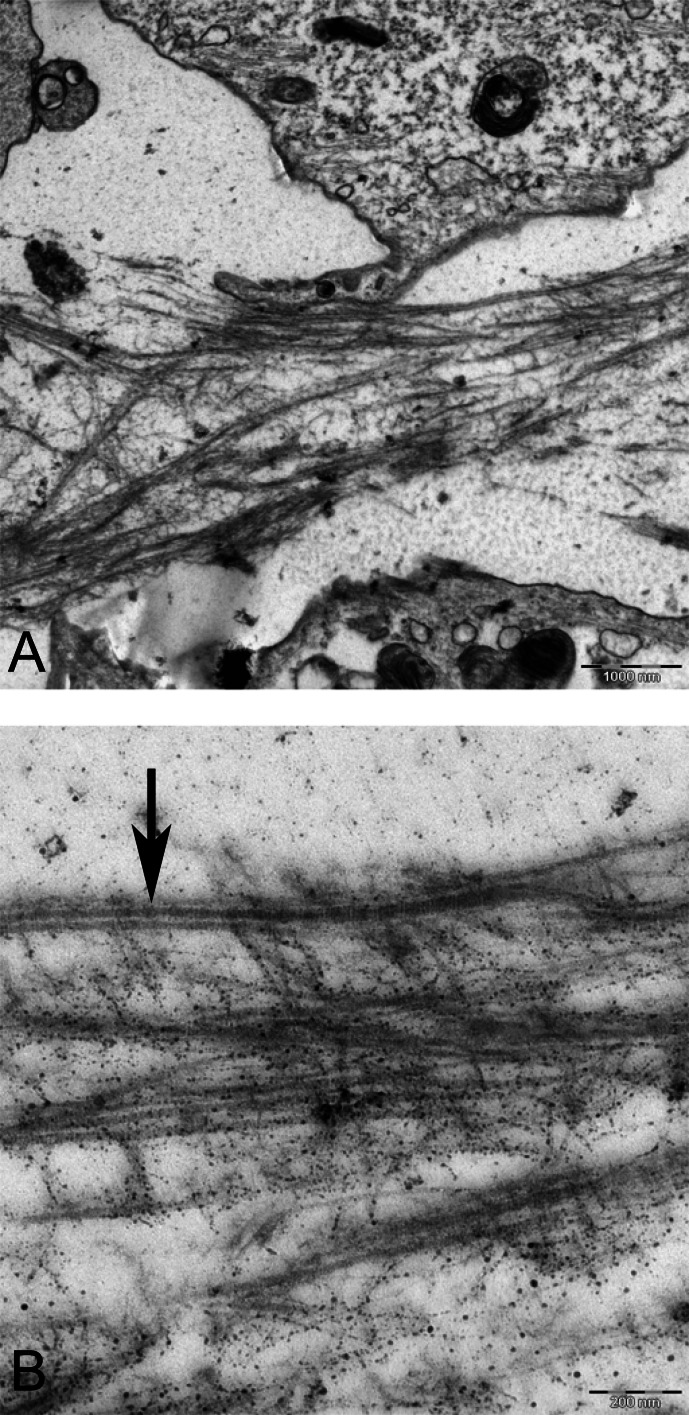
TEM images of extracellular matrix of MSCs in pellet culture after 21 days in chondrogenic medium. (A) Collagen fibres (F) in the ECM 2500X. (B) Higher magnification 10000X shows striation (arrow) on fibres.

Two subpopulations of cells can be distinguished in ultrathin sections of pellet culture; In one group, signs of cell death appeared with abundant cytoplasmic vesicles, lipid droplets, free ribosomes, swelled or fused mitochondria (Fig. 13A), vacuoles, expelling of cytoplasmic organelles to ECM (Fig. 13B), indented nucleus (Fig. 13D), or cells without a prominent nucleus (Fig. 13C). The second population consisted of active protein-synthesizing cells with a euchromatin and round nucleus and abundance of collagen fibers secreted in the ECM (Figs. 14 and 15). Golgi apparatus can be observed in both groups (Figs. 13D and 14A) as machinery for synthesis of carbohydrates (Alberts et al., 2002) or GAGs in chondrogenic-induced MSCs.

## Discussion

Gene expression relating to chondrogenic markers, which include Collagen type II and Sox 9, and aggrecan in alginate beads in this study was found to be higher than that of pellet or monolayer culture. This is due to the fact that newly formed chondrogenic construct in alginate culture resembles an immature fetal cartilage in which anabolic activity surpluses the catabolic activity in chondrocytes and results in a higher production of ECM, even when compared with an adult cartilage tissue (Aigner, Soeder & Haag, 2006).

These results suggest that chondrogenic differentiation of MSCs in 3D culture, i.e., alginate, is superior as compared with pellet and 2D monolayer cultures. Our results are consistent with those of previous researches (Yang et al., 2004) but contradict others (Ichinose et al., 2005). In several studies, chondrogenic differentiation of MSCs in alginate beads induced with TGF-*β*3 calcification was reported. In our study, hypertrophic genes Runx2 and Col X were downregulated in alginate group but they were upregulated in pellet and monolayer cultures using similar culture medium.

Expression of transcription factor Sox 9 is accompanied by the expression of chondrogenic gene, such as Col2a1, and aggrecan (De Crombrugghe et al., 2000). In this study, increase of expression of Sox 9 overlaps with the expression of Col 2 and aggrecan genes in 3D chondrogenic systems. However, in monolayer cultures, it was not accompanied by an increase in aggrecan. In this experiment, expression of Sox 9 remained low in monolayer and chondrogenic pellet culture models, whereas the expressions of Col X and Runx2 were obvious in these groups. Considering the fact that Sox 9 inhibits hypertrophic changes in chondrocytes (Murakami et al., 2004; Ikegami et al., 2011; Dy et al., 2012) in this study, higher expression of Sox 9 in alginate culture may have protected the cells from being differentiated to a hypertrophic state.

Cell adhesion molecules N-CAM1 and N-cadherin were found to be downregulated in 3D cultures of alginate beads and pellet cultures on day 3. These results are consistent with downregulation of these genes during embryonic chondrogenesis (Widelitz et al., 1993; Tavella et al., 1994), in which N-CAM1 and N-cadherin downregulated after the expression of chondrocyte-specific genes. On the other hand, during chondrogenic differentiation of MSCs in periosteal membranous bone in craniofacial skeleton such as avian quadratojugal joint (an equivalent to mammalian mandibular condylar cartilage), it was shown that N-CAM was not necessary before chondrogenesis (Fang & Hall, 1999). MSCs in gel-like biomaterials, such as Col I, fibrin glue, Matrigel, and PuraMatrix peptide hydrogel, underwent proper chondrogenesis without a direct cell–cell communication (Dickhut et al., 2008). Therefore, it seems that direct cell–cell interactions both *in vitro* and *in vivo* are not always necessary for chondrogenic differentiation (Boeuf & Richter, 2008). However, indirect paracrine communication between the cells in the gel-like material might play a role as it has been shown that MSCs express cytokines and growth factors (Kim et al., 2005).

N-cadherin remained downregulated in both groups of pellet and alginate cultures until third week of differentiation and was later expressed in both groups on day 21. In a limb bud experiment, it was shown that N-cadherin was expressed before chondrogenesis in condensed MSCs and then disappeared from the center of condensation while the cells continued their differentiation and then reexpressed in perichondrium, an indication of appositional growth (Oberlender & Tuan, 1994). As a comparison, the expression of N-cadherin in our experiment on day 21 can be justified with appositional growth of chondrogenic model of pellet in which the peripheral undifferentiated layer of cells behave as perichondrium (Hillel et al., 2010) or fibroblast (Alberts et al., 2002).

TEM analysis revealed prevalence of cell death in pellet culture, as compared with alginate culture. It can be due to an immature hypertrophy and cell death, as it may happen during endochondral ossification (Adams & Shapiro, 2002; Mackie et al., 2008). The type of cell death can be nonapoptotic or physiologic cell death because it lacked the whole characterization of apoptotic cells, such as crescent heterochromatin nucleus (Zamli & Sharif, 2011). However, there is a possibility that distinctive morphology of apoptotic nucleus is missing in our experiment due to its occurrence in an earlier time point.

The morphology and abundance of mitochondria in MSCs and alginate culture were found to be similar (Figs. 8 and 11). Hypoxic microenvironment in cartilage may have caused lower number of mitochondria in articular cartilage as compared with metabolically active cells because chondrocytes rely on glycolytic metabolism rather than oxidative phosphorylation (Milner, Wilkins & Gibson, 2012). In this study, we did not perform any quantitative method for comparing mitochondria in MSC and CMSC; however, the qualitative assessment using pictures did not show any difference in mitochondrial density between undifferentiated MSCs and chondrogenic MSCs. This may be because culture conditions at ambient oxygen of 20% do not provide similar conditions to normal habitat of chondrocyte in cartilage, which is usually low at an oxygen concentration of 2–10% (Zhou, Cui & Urban, 2004). It has been shown that chondrocytes were isolated from joint and cultured *in vitro* expressed mitochondrial biosynthesis (Milner, Wilkins & Gibson, 2012). Further studies using immunofluorescence for detecting mitochondria can verify mitochondrial quantities during *in vitro* chondrogenic differentiation of MSCs. The swollen and fused mitochondria from the pellet culture might be due to hypertrophy and cell death. Mitochondrial fusion was shown to be accompanied with MSCs undergoing cell death. Fusion of mitochondria is described as a reaction of the cells to damaged mitochondria to repair by intermixing DNA and protein between mitochondria during damage or senescence (Chan, 2006). Swollen mitochondria were also reported in fibrillated cartilage in OA patients (Roy & Meachim, 1968).

The morphology of nucleus in alginate and pellet cultures varied. It appears that cells are more elongated and indented in pellet, whereas in alginate, cells were mostly oval and spherical. Although a typical chondrocyte may have a spherical nucleus, previous ultrastructural studies demonstrated that variation in shapes including oval, spherical, elongated, or indented in human articular cartilage is common, which appears to be acceptable for chondrocyte morphology (Roy & Meachim, 1968).

Although the study conducted was well designed, several limitations need to be mentioned to ensure that the findings of the study are not overstated. One of the limitations is that of the time points used for the investigation. The study is only limited to three weeks, which may not be sufficient to demonstrate hypertrophy in our alginate cultures because there has been at least one study that demonstrated that hypertrophy may need up to 45 days of culture (Dickhut et al., 2008). The second limitation of the study is the fact that the study was limited to gene expression and selected protein investigations. The use of other investigative tools, which includes Western blotting, or protein profile of the constructs would have strengthened the paper further. This is especially true for hypertrophic markers, such as Collagen X and Runx2 and adhesion molecules, such as N-CAM1 and N-cadherin.

## Conclusion

The present study suggests that alginate bead culture provides superior chondrogenic differentiation while decreasing the hypertrophic markers as compared with pellet and monolayer cultures. Alginate bead cultures resemble the articulate cartilage model, whereas pellet cultures resemble the endochondral ossification; therefore, alginate culture would be more suited as an articular cartilage model, whereas the pellet culture would be more appropriate for hypertrophic model of chondrogenesis.

## Supplemental Information

10.7717/peerj.1650/supp-1 Data S1 Raw data

## Additional Information and Declarations

### Data Availability

The following information was supplied regarding data availability: Data S1 has been provided in the Supplemental Information.
